# Diagnostic Accuracy of Cone-Beam Computed Tomography and Periapical Radiography in Internal Root Resorption

**DOI:** 10.7508/iej.2016.01.010

**Published:** 2015-12-24

**Authors:** Zahrasadat Madani, Ehsan Moudi, Ali Bijani, Elham Mahmoudi

**Affiliations:** a * Department of Endodontics, Dental School, Babol University of Medical Sciences, Babol, Iran; *; b *Department of Oral and Maxillofacial Radiology, Dental School, Babol University of Medical Sciences, Babol, Iran; *; c *Social Determinants of Health Research Center, Babol University of Medical Sciences Babol, Iran*

**Keywords:** Cone-Beam Computed Tomography, Periapical Radiography, Root Resorption

## Abstract

**Introduction:**

The aim of this study was to compare the diagnostic value of cone-beam computed tomography (CBCT) and periapical (PA) radiography in detecting internal root resorption.

**Methods and Materials:**

Eighty single rooted human teeth with visible pulps in PA radiography were split mesiodistally along the coronal plane. Internal resorption like lesions were created in three areas (cervical, middle and apical) in labial wall of the canals in different diameters. PA radiography and CBCT images were taken from each tooth. Two observers examined the radiographs and CBCT images to evaluate the presence of resorption cavities. The data were statistically analyzed and degree of agreement was calculated using Cohen’s kappa (k) values.

**Results:**

The mean±SD of agreement coefficient of kappa between the two observers of the CBCT images was calculated to be 0.681±0.047. The coefficients for the direct, mesial and distal PA radiography were 0.405±0.059, 0.421±0.060 and 0.432±0.056, respectively (*P*=0.001). The differences in the diagnostic accuracy of resorption of different sizes were statistically significant (*P*<0.05); however, the PA radiography and CBCT, had no statistically significant differences in detection of internal resorption lesions in the cervical, middle and apical regions.

**Conclusion:**

Though, CBCT has a higher sensitivity, specificity, positive predictive value and negative predictive value in comparison with conventional radiography, this difference was not significant.

## Introduction

Internal resorption is the progressive damage of intracanal dentin due to the multinucleated giant cells adjacent to the granulated tissue in the pulp. Except for the pulpectomized primary teeth, internal resorption is considered as a rare pathology which occurs due to inflammation or infection within the canal or trauma to the tooth, when the protective coat of predentin is damaged and the activity of clastic cells leads to resorption in a way that the recessed space would be filled with granulation tissue or in combination with a bone-like or cementum-like mineralized tissue [1, 2].

These mechanisms were first described in 1829 [3]; however, the etiologies and pathogenesis are not completely understood yet. In comparison with external resorption, internal resorption rarely occurs and it also has a totally different treatment approach. Although internal resorption often affects the cervical area of the root, it can occur in any region [4]. This phenomenon is highly prevalent in the teeth under special treatments such as auto-transplant procedure. 

Internal resorption usually has no sign and in a routine radiography it is recognized as a uniform enlargement of the pulp canal. If it is diagnosed and treated at an early stage, its propagation and subsequent perforation could be prevented [5]. In case of perforation the prognosis would be poor [6]. For diagnosis of these lesions, the conventional or digital radiography is usually helpful [7, 8]. 

Up to the present, the diagnostic accuracy of digital radiography for the evaluation of dental cavities [9], measurement of the endodontic variables [10], measurements of periapical lesions [11], root cracks [12-14] and assessment of implants [15] has been established. Furthermore, the capability of digital sensors in the diagnosis of external [16, 17] and internal [18] root resorption has been investigated. In teeth with external resorption, the radiolucent regions are observed in radiographic images that move in different horizontal angles in angled radiographs, but in internal resorption lesions, it remains near the root canal [19]. Due to their characteristics, intraoral techniques, show three-dimensional (3D) structures as a two-dimensional image. As a result, radiographic images offer limited amount of information to technicians regarding the size of lesion and its level of extension. Also, the diagnostic accuracy of the intraoral radiographs is affected by anatomic superimpositions, angle of the x-ray spectrum and image preparation steps [20]. 

A 3D imaging system could be useful in making a faster diagnosis of resorption lesions; for instance when resorption is suspected after trauma, more precise information could be provided by 3D imaging about the type of resorption (either external or internal), the location of resorption (either cervical, mid-root or apical), its size and proximity to the canal or periodontal space facilitating the proper and adequate treatment [21].

In medicine, computed tomography (CT) is known as the main diagnostic device due to its ability to present useful information about the anatomy of bony structures [22, 23]. However, the technical problems associated with CT systems such as high exposure doses and high costs, low resolution and *etc*., have made it an undesirable method in routine dental imaging [24]. Cone-beam computed tomography (CBCT) is another method, which has made a great advancement in dental, oral and maxillofacial imaging technology, in a way that it provides a cross-sectional 3D image with less radiations dose, shorter scanning time, less artifacts and lower price in comparison with conventional CT. This method has been widely used as a diagnostic tool in many cases before surgery [25, 26].

Ezzodini *et al.* [12] investigated the accuracy of two imaging methods (CBCT and PA radiography) in detecting vertical root fractures. The results showed that the overall sensitivity of CBCT was significantly higher than PA radiography. Vast amount of evidence exist on the efficiency of CBCT in the diagnosis and treatment of the external and internal root resorption; but still intraoral PA radiographies have been widely applied in the diagnosis and treatment of the resorptions. Until now, only a few researches have conducted comparative studies regarding the diagnostic value of CBCT and PA radiography in the diagnosis of the internal resorption [18, 19]. 

The aim of this *in vitro *study was to compare the diagnostic value of PA radiography and CBCT in different types of resorptive lesions, in different places and with variable sizes to determine an appropriate indication for CBCT in suspicious cases.

## Materials and Methods:

This experimental *in vitro *study was done on 80 single rooted extracted human teeth. For disinfection, the teeth were placed in 2% glutaraldehyde solution [27], and then were maintained in the normal saline. In radiography, the teeth had non-calcified canals and no anomalies or root fillings. The teeth were split into buccal and lingual halves by a diamond disk in a mesiodistal plane. The teeth were then divided into 7 experimental groups (*n*=10), and one control group with 10 teeth which had no resorption cavities. 

Depending on the group, the resorption cavities were created in the cervical, middle and apical regions or a combination of them on the internal wall of the labial half of the teeth. Then the groups were divided into three subgroups and the cavities were created in the middle and apical regions. In the first subgroup (4 samples) the resorption cavities were formed in two regions of the middle and apical areas by round bur (SS White Burs Inc., Lakewood, NJ, USA) with a diameter of 0.6 mm at half of its depth. In the second subgroup (3 samples), the resorption cavities were formed in the same areas by round bur with a diameter of 0.8 mm at half of its depth; also resorption cavities in the third subgroup (3 samples) were created by round bur with a diameter of 1.2 mm at half of its depth. In the next groups, the resorption process was carried out in the similar manner (Table 1).

Finally, the two buccal and lingual halves of the tooth were joined together by super glue and then the roots of the teeth were covered by a wax layer to reduce the artifact [15]. The teeth were mounted on a mixture containing equal ratios of plaster and ground rice [22]. 

**Table 1:** The characteristic of groups

**Group (N) **	**Location of internal resorption**		
	**Apical**	**Middle**	**Cervical**
**Group 1 (10)**	-	-	-
**Group 2 (10)**	+	+	-
**Group 3 (10)**	-	+	-
**Group 4 (10)**	+	-	-
**Group 5 (10)**	+	+	+
**Group 6 (10)**	-	-	+
**Group 7 (10)**	-	+	+
**Group 8 (10)**	+	-	+

To minimize the errors in the interpretation of the images, the process of sample selection and creation of the resorption was done by the researcher and then in order to blind the study; the samples were coded by a person outside the study and presented to the observers. The radiographic images were taken by means of E-speed Kodak film size 2 (Eastman-Kodak Co., Rochester, NY, USA) and the x-ray device of MinRay (Sordex, Helsinky, Finland) with exposure settings of 60 kVp, 7 mA and 0.2 sec. These images were prepared in three direct, mesial and distal planes with 20-degree differences. The processing of these films was done by an automatic film processor (HOPE Dental-max, Hoop Co., USA).

**Figure 1:**
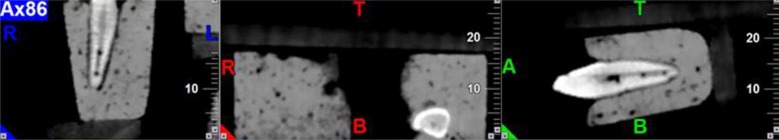
Multi-planar sectional images of simulated internal root resorption lesions

The CBCT images were prepared using NewTom VG 9000 CBCT device (Quantitative Radiology SRL Co., Verona, Italy) with a 6×6 cm field of view (FOV) under the standard conditions. The CBCT images were reconstructed and investigated in three planes: axial, coronal, sagittal and MPR using NTT Viewer software program (NTT Software Corporation, Yokohama, Japan) (Figure1). The prepared films and also the CBCT images on the monitor were blindly investigated twice by an oral and maxillofacial radiologist and an endodontist. The level of agreement between the observers was analyzed using Cohen’s kappa (k) coefficient values. Then the data were qualitatively and quantitatively analyzed by CAT MAKER. Sensitivity, specificity, positive predictive value and negative predictive value and the positive and negative likelihood ratio were calculated by kappa index. The level of significance was set at 0.05.

## Results

A total of 120 resorption-like sites were created on 240 available sites of the sample teeth (Table 1). The kappa coefficient value between the two observers was calculated as 0.681±0.047 for CBCT images. Also, the kappa coefficients for the two observers regarding their decision on direct-, mesial- and distal-angled radiographies with 20 degrees difference were 0.405±0.059, 0.421±0.060 and 0.432±0.056, respectively (*P*=0.001).

The results of sensitivity, specificity, positive predictive value and negative predictive value of CBCT images and the PA radiography in three direct, mesial and distal angles with a 20-degree difference among the study samples are shown in Table 2. Sensitivity, specificity, positive predictive value, and negative predictive value of CBCT images were 88, 76, 79 and 87 percents, respectively; which were higher than the results obtained from PA radiography.

The difference in the diagnostic accuracy of resorption lesions with different sizes (half depth of 0.6, 0.8 and 1.2 mm burs) were statically significant (*P*<0.05); which means that the larger lesions could be better diagnosed in both methods of PA radiography and CBCT (Table 3). But for the diagnosis of internal resorption lesions in the three cervical, middle and apical regions, no statistically significant difference was observed; the PA radiography nor the CBCT images had similar diagnostic accuracy in detecting internal root resorption.

**Table 2:** Percent (range) of sensitivity, specificity, positive predictive value (PPV), and negative predictive value (NPV), positive likelihood ratio (LR+) and negative likelihood ratio (LR-) (confidence interval, CI=95%)

**Imaging (observer 1 or 2)**	**Sensitivity**	**Specificity**	**PPV**	**NPV**	**LR+**	**LR-**
**CBCT (1)**	86 (80-92)	80 (73-87)	81 (74-88)	85 (78-92)	4.29 (2.98-6.18)	0.18 (0.11-0.28)
**CBCT** **(2)**	88 (83-94)	76 (68-83)	79 (72-85)	87 (80-93)	3.66 (2.64-5.05)	0.15 (0.09-0.25)
**PA radiography** **(1)**	47 (38-56)	73 (65-81)	64 (54-74)	58 (50-66)	1.75 (1.23-2.49)	0.73 (0.60-0.89)
**PA radiography** **(2)**	64 (56-73)	77 (69-84)	73 (65-82)	68 (60-76)	2.75 (1.94-3.91)	0.47 (0.36-0.61)
**Mesial-angled radiography** **(1)**	54 (45-63)	73 (65-81)	67 (58-76)	62 (54-70)	2.03 (1.45-2.85)	0.63 (0.50-0.78)
**Mesial-angled radiography** **(2)**	61 (52-70)	79 (72-86)	74 (66-83)	67 (59-75)	2.92 (2.00-4.26)	0.49 (0.39-0.63)
**Distal-angled radiography** **(1)**	59 (50-68)	77 (69-84)	72 (63-81)	65 (57-73)	2.54 (1.77-3.62)	0.53 (0.42-0.67)
**Distal-angled radiography** **(2)**	63 (55-72)	73 (65-81)	70 (62-79)	67 (59-75)	2.38 (1.71-3.29)	0.50 (0.39-0.65)

**Table 3:** Diagnostic accuracy [N(%)] of internal resorption with different sizes

**Imaging (observer 1 or 2)**	**Recession size (N)**					
	**0.0 (120)**	**0.3 mm (48)**	**0.4 mm (36)**	**0.6 mm (36)**	***P*** **-value**	
**CBCT (1)**	96 (80)	40 (83.3)	28 (77.8)	35 (97.2)	0.086
**CBCT** **(2)**	91 (75.8)	37 (77.4)	34 (94.4)	35 (97.2)	0.004
**PA radiography** **(1)**	92 (76.7)	22 (45.8)	24 (66.7)	25 (69.4)	0.002
**PA radiography** **(2)**	88 (73.3)	35 (72.9)	17 (47.2)	24 (66.7)	0.025
**Mesial-angled radiography** **(1)**	88 (73.3)	18 (37.5)	23 (63.9)	24 (66.7)	<0.001
**Mesial-angled radiography** **(2)**	95 (79.2)	28 (58.3)	20 (55.6)	25 (69.4)	0.009
**Distal-angled radiography** **(1)**	88 (73.3)	17 (35.4)	17 (47.2)	22 (61.6)	<0.001
**Distal-angled radiography** **(2)**	92 (76.7)	28 (58.3)	22 (61.1)	27 (75)	0.058

## Discussion

In the presented study, the diagnostic accuracy and value of the PA radiography and CBCT were evaluated and their capabilities in guiding to a correct diagnosis were investigated. According to the results CBCT had a higher accuracy in detection of internal resorption in comparison with the PA radiography, however the differences were not significant.

The obtained results by the current study are in accordance with the results obtained by Kamburoglu* et al*. [28]. In their study the kappa agreement value was 70 and 40% for CBCT and radiography, respectively [28]. It was also in agreement with the results of the study by Ozen *et al.* [29] stating that CBCT is better than PA images in the diagnosis of periapical lesions. Also another study by Kamburoglu *et al.* [19] investigated the diagnostic accuracy of CBCT with different resolutions in identifying internal resorption. Moreover, in their study, the amount of KAPA agreement coefficient between the observers was fair to moderate regarding images with a low resolution, and in images with high resolution, this parameter was reported as good to excellent.

In the present study, the differences between the diagnostic accuracy of resorption lesions in different sizes were statistically significant (*P*<0.05); this means that the larger lesions would be better diagnosed in both methods of PA radiography and CBCT. In the study by Kamburoglu *et al.* [18], they compared the conventional, digital and digitally filtered images in the diagnosis of internal resorption in human skull and came to the conclusion that the apically located resorptions are the most difficult ones to diagnose, and the resorption in cervical regions had a higher ratio of accurate diagnosis. Also in their study they found that the increase in the size of damage could increase the diagnostic ability [18]. Our results did not show a significant difference in the position of the damage; which were compatible with the results obtained by Anderson *et al*. [30] who reported no significant difference in the diagnosis of resorption in the cervical, middle or apical regions. However this is in contrast with the results reported by Kamburoglu *et al*. [18] who conducted their research on the mandibular anterior teeth with a small pulp canal in comparison with the aforementioned study which had used maxillary central incisors; therefore it can be concluded that the diagnosis of the internal resorption in the apical regions is significantly more difficult. In the present study, the teeth were dominantly the anterior maxillary and the mandibular premolars.

Patel *et al*. [6], conducted their research on patients with internal and external resorption, and concluded that although the PA radiography is an acceptable diagnostic device, CBCT had more precision in the diagnosis of the internal resorption and consequently this method could enhance the chance and probability of a correct treatment.

In another similar study, the inflammatory resorption of the root were assessed by means of CBCT in the apical, middle, and cervical regions, from mesial, distal, buccal, palatal, lingual, and apical aspects: inflammatory resorption of the root was observed in 68.8% of the radiographies and 100% of the CBCT images. In addition, it was observed that the expansion of inflammatory resorption of the root was larger than 1-4 mm in 95.8% of CBCT images and 52.1% of conventional radiographic images. As a result, it was concluded that the expansion of resorption could be diagnosed by the CBCT more precisely and in earlier stages [31].

The superiority of CBCT is attributed to the 3D nature of the images it obtains from the target region [6]. Moreover, it offers various options and settings in the field of image thickness and provides the desired view for the radiologist to gain a better understanding of the internal resorption [18]. The introduction of 3D imaging was initially made for dealing with the limitations of conventional imaging systems. For instance, Friedland *et al.* [32] suggested that CBCT could be regarded as a suitable method for imaging the buccolingual view of teeth affected by internal resorption. Researchers believed that the addition of one more dimension to radiographic images could significantly enhance the trend in the diagnosis of resorption [33]. Trope *et al.* [34] reported that the frequency of correct treatment decisions made for resorption obtained from CBCT was significantly higher than those deducted from intraoral radiographic methods. Ahlowalia *et al*. [35] conducted a research aimed to investigate the accuracy of CBCT in measurement of the different aspects of PA lesions on a cow bone model; they showed that both CBCT and Micro-CT led to the close-to-real results.

Moreover, in recent studies, the effect of factors such as size, voxel and type of filter enhancement used in different CBCT devices have been studied regarding their diagnostic capability for observers [27, 36, 37]. In one study which compared the diagnostic accuracy of CBCT images in different voxel resolutions for detection of the internal resorption, it was shown that in high resolutions the two devices worked similarly and their efficiency was more with low resolution [19]. The efficiency of CBCT was also investigated in another similar study which aimed at comparing devices such as CBCT and digital intraoral radiography for diagnosis of the vertical root fracture. It was revealed that in higher resolutions, both devices had higher efficiency in comparison with lower resolutions and intraoral digital radiography [38]. These results were in agreement with a similar research performed on the internal and external resorption formed in cervical regions of the root [28]. The size and voxel are also among the parameters that play an important role in the quality and reconstruction of the scans. The effect of voxel size on the diagnostic capability of CBCT for evaluating internal [19] and external [39] root resorption has been established. In conclusion, the cavities created by a round bur have more defined borders than natural shapes which make diagnosis easier [18]; as a consequence, this suggests further studies to be carried out to investigate the natural forms of resorption lesions.

## Conclusion

In comparison with conventional radiography, CBCT has a higher sensitivity, specificity, positive predictive value and negative predictive value in diagnosis of internal root resorption. However this difference is not significant.
